# Association between modified CHA_2_DS_2_-VASc Score with Ankle-Brachial index < 0.9

**DOI:** 10.1038/s41598-018-19243-y

**Published:** 2018-01-19

**Authors:** Po-Chao Hsu, Wen-Hsien Lee, Hsiang-Chun Lee, Wei-Chung Tsai, Chun-Yuan Chu, Ying-Chih Chen, Chee-Siong Lee, Tsung-Hsien Lin, Wen-Chol Voon, Sheng-Hsiung Sheu, Ho-Ming Su

**Affiliations:** 1 Division of Cardiology, Department of Internal Medicine, Kaohsiung Medical University Hospital, Kaohsiung Medical University, Kaohsiung, Taiwan; 2 Faculty of Medicine, College of Medicine, Kaohsiung Medical University, Kaohsiung, Taiwan; 3 Department of Internal Medicine, Kaohsiung Municipal Hsiao-Kang Hospital, Kaohsiung, Taiwan

## Abstract

The ankle-brachial index (ABI) is a reliable diagnostic examination for peripheral arterial occlusive disease (PAOD). We previously reported CHADS_2_ score was significantly correlated with PAOD. However, the association between CHA_2_DS_2_-VASc score and ABI < 0.9 is not evaluated in the literature. The aim of the present study was to investigate whether CHA_2_DS_2_-VASc score has a strong association with PAOD. We enrolled 1482 patients in this study. PAOD was defined as ABI < 0.9 in either leg. Vascular disease in CHA_2_DS_2_-VASc score was modified as vascular disease except PAOD. Of the 1482 subjects, the prevalence of ABI < 0.9 was 5.6%. Multivariate analysis showed that the increased age, decreased estimated glomerular filtration rate and increased modified CHA_2_DS_2_-VASc score (OR, 1.764; p < 0.001) were independent associated with ABI < 0.9. In addition, the percentage of ABI < 0.9 in patients with modified CHA_2_DS_2_-VASc score of 0, 1, and <2 were 0%, 0.9%, and 0.7%, respectively (All < 1%). Our study demonstrated modified CHA_2_DS_2_-VASc score was significantly associated with ABI < 0.9. Calculation of modified CHA_2_DS_2_-VASc score might be useful in identifying patients with PAOD and in stratifying the risk of PAOD in non-AF patients.

## Introduction

The ankle-brachial index (ABI) is a non-invasive and reliable diagnostic examination for peripheral arterial occlusive disease (PAOD)^1,2^. An ABI < 0.9 has not only been regarded as a reliable diagnostic tool for PAOD, but also a strong predictor for overall and cardiovascular mortality in different populations^3–5^.

PAOD shares similar major risk factors with coronary artery disease (CAD) and cerebrovascular disease^6^. Patients with one vascular bed disease often have coexistent diseases in other vascular beds^7^. Patients with PAOD have increased cardiovascular morbidity and mortality especially for patients with critical limb ischemia^8,9^. Major risk factors for PAOD include advanced age, hypertension, diabetes mellitus, dyslipidemia, and smoking^6,10^. In addition, stroke, CAD, chronic kidney disease, races, gender, and heart failure were also reported to be associated with PAOD formation^11–18^.

In our previous study, we found an association between CHADS_2_ (congestive heart failure, hypertension, age ≧75 years, diabetes, prior stroke) score and ABI < 0.9 and confirmed CHADS_2_ score was significantly associated with ABI < 0.9 in non-atrial fibrillation (AF) patients^19^. Additionally, using our National Health Insurance Research Dataset, we further demonstrated that CHADS_2_ score was useful in predicting the risk of new-onset PAOD^20^. The CHADS_2_ score is a simple and popular clinical score to assess the risk of stroke in patients with AF and there is a direct relationship between the CHADS_2_ score and the annual risk of stroke in AF patients^21^. However, CHADS_2_ score has several limitations. For example, some stroke risk factors were not included and patients with CHADS_2_ score of 0 still had annual stroke rate >1.5%. Therefore, CHADS_2_ score of 0 may not identify AF patients as low risk of stroke reliably. In recent years, CHA_2_DS_2_-VASc score has become a more popular score to evaluate the annual risk of stroke in AF patients^22–26^. This score included more stroke risk factors and could identify the low stroke risk AF patients who did not need antithrombotic therapy^22–26^. It had also been validated in Asian population as a more reliable score system than CHADS_2_ score for the assessment of stroke risk in AF patients^27–29^.

Because the major cardiovascular risk factors affect all vascular territories, it means that patients with one vascular territory disease are more susceptible to have another vascular territory disease. Therefore, CHA_2_DS_2_-VASc score should have a significant correlation with PAOD. Here, we designed the study to investigate whether CHA_2_DS_2_-VASc score was significantly associated with PAOD confirmed by ABI < 0.9 and evaluate if this score could help physicians to further stratify the risk of PAOD.

## Results

The prevalence of ABI < 0.9 was 5.6%. Table 1 shows the comparison of clinical characteristics between patients with and without ABI < 0.9. The mean age of the 1482 patients was 61.4 ± 13.6 years. Compared with patients with ABI ≥ 0.9, patients with ABI < 0.9 were found to have an older age, higher prevalence of heart failure, hypertension, diabetes, cerebrovascular disease, and CAD, higher CHADS_2_ score, higher modified CHA_2_DS_2_-VASc score, lower BMI, lower eGFR, higher uric acid level, and higher percentage of using of aspirin, ARBs, diuretics, and statins.

**Table 1:** Comparison of clinical characteristics between patients with ABI < 0.9 and ≥0.9.

Characteristics	All patients (n = 1482)	ABI < 0.9 (n = 83)	ABI ≥ 0.9 (n = 1399)	P value
Age (year)	61.4 ± 13.6	73.1 ± 13.3	60.7 ± 13.3	<0.001
Male gender (%)	56.5	55.4	56.5	0.909
Smoking history (%)	15.2	11	15.5	0.400
Heart failure (%)	8.9	25.3	7.9	<0.001
Hypertension (%)	70.0	84.3	69.1	0.003
Diabetes Mellitus (%)	28.7	59.0	26.9	<0.001
Cerebrovascular disease (%)	6.1	15.7	5.5	0.001
CAD (%)	16.6	30.1	15.8	0.002
CHADS2 score	1.38 ± 1.06	2.58 ± 1.14	1.30 ± 1.01	<0.001
CHA2DS2-VASc score	2.40 ± 1.48	4.12 ± 1.44	2.30 ± 1.41	<0.001
BMI	26.1 ± 3.98	24.6 ± 3.63	26.2 ± 3.98	<0.001
**Laboratory parameters**				
Triglyceride (mg/dl)	153.0 ± 145.0	151.1 ± 91.3	153.2 ± 147.2	0.866
Total cholesterol (mg/dl)	191.4 ± 43.3	195.0 ± 50.1	191.2 ± 42.0	0.510
Uric acid (mg/dl)	6.9 ± 2.1	7.6 ± 2.2	6.8 ± 2.1	0.015
eGFR (ml/min/1.73 m^2^)	57.2 ± 21.1	38.5 ± 19.5	58.3 ± 20.7	<0.001
**Medications**				
Aspirin use (%)	32.4	47.9	31.4	0.004
ACEI use (%)	11.6	18.3	11.2	0.073
ARB use (%)	43.3	55.4	42.6	0.023
CCB use (%)	36.7	42.2	36.4	0.293
β-blocker use (%)	40.8	45.8	40.5	0.359
Diuretic use (%)	28.9	48.2	27.8	<0.001
Statin use (%)	18.0	28.8	17.4	0.018

Abbreviations: ABI, ankle-brachial index; eGFR, estimated glomerular filtration rate; ACEI, angiotensin converting enzyme inhibitor; ARB, angiotensin II receptor blocker; CAD, coronary artery disease; CCB, calcium channel blocker.

Table 2 shows the determinants of ABI < 0.9 in all study patients. In the univariate analysis, ABI < 0.9 was found to be significantly associated with increased age, a history of heart failure, hypertension, diabetes, cerebrovascular disease, and coronary artery disease, low BMI, high CHADS_2_ score, high modified CHA_2_DS_2_-VASc score, low eGFR, high uric acid level, and high percentage of using aspirin, ARBs, diuretics, and statins. In the forward multivariate logistic analysis, increased age (odds ratio [OR], 1.058; 95% confidence interval [CI], 1.017–1.101; p = 0.005), lower eGFR (OR, 0.977; 95% CI, 0.960–0.994; p = 0.01), and high modified CHA_2_DS_2_-VASc score (OR, 1.764; 95% CI, 1.338–2.325; p < 0.001) were associated with ABI < 0.9.

**Table 2:** Determinants of ABI < 0.9 in study patients.

Parameter	Univariate		Multivariate (Forward)	
	OR (95% CI)	*P*	OR (95% CI)	*P*
Age (per 1 year)	1.088 (1.065–1.111)	<0.001	1.058 (1.017–1.101)	0.005
Male gender	1.046 (0.670–1.634)	0.842	—	—
Smoking (ever *versus* never)	0.671 (0.316–1.421)	0.297	—	—
Diabetes mellitus	3.921 (2.492–6.169)	<0.001	—	—
Hypertension	2.406 (1.317–4.395)	0.004	—	—
Congestive heart failure	3.930 (2.310–6.687)	<0.001		
Cerebrovascular disease	3.188 (1.690–6.017)	<0.001	—	—
Coronary artery disease	2.294 (1.405–3.747)	0.001	—	—
Body mass index (per 1 kg/m^2^)	0.891 (0.836–0.949)	<0.001	—	—
CHADS2 score	2.539 (2.097–3.074)	<0.001	—	—
CHA2DS2-VASc score	2.127 (1.826–2.477)	<0.001	1.764 (1.338–2.325)	<0.001
eGFR (per 1 mL/min/1.73 m^2^)	0.961 (0.951–0.971)	<0.001	0.977 (0.960–0.994)	0.010
Laboratory parameters				
Triglyceride (mg/dL)	1.000 (0.998–1.002)	0.866	—	—
Total cholesterol (mg/dL)	1.002 (0.996–1.008)	0.509	—	—
Uric acid (mg/dL)	1.150 (1.027–1.288)	0.015	—	—
Medications				
Aspirin use	2.012 (1.251–3.236)	0.004	—	—
ACEI use	1.768 (0.986–3.171)	0.056	—	—
ARB use	1.676 (1.073–2.617)	0.023	—	—
β-blocker use	1.238 (0.794–1.932)	0.346	—	—
CCB use	1.276 (0.814–1.999)	0.287	—	—
Diuretic use	2.417 (1.547–3.777)	<0.001	—	—
Statin use	1.922 (1.133–3.262)	0.015	—	—

Values expressed as odds ratio (OR) and 95% confidence interval (CI). Abbreviations are the same as in Table 1.

**Figure 1:**
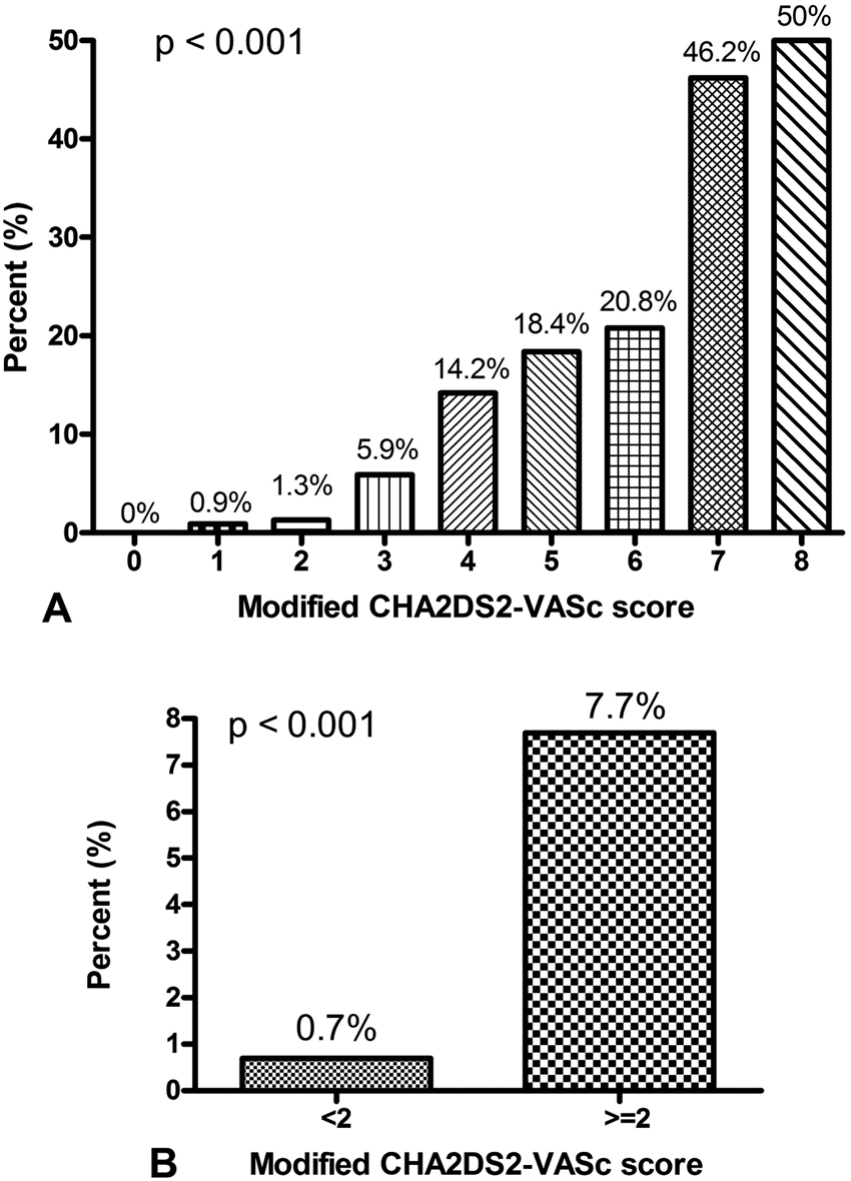
The percentage of ABI < 0.9 in patients with different modified CHA_2_DS_2_-VASc score (**A**) and in patients with modified CHA_2_DS_2_-VASc score <2 and ≥2 (**B**).

The percentage of ABI < 0.9 in patients with CHADS_2_ score of 0, 1, 2, 3, 4, and 5 was 0.7%, 1.7%, 7.5%, 19.7%, 21.4%, and 37.5%, respectively (p < 0.001). There was no patient with CHADS_2_ score of 6 in our study. The percentage of ABI < 0.9 in patients with CHADS_2_ score of <2 and ≥2 was 1.3% and 12.0%, respectively (p < 0.001). Figure 1A shows the percentage of ABI < 0.9 in patients with different modified CHA_2_DS_2_-VASc score. The percentage of ABI < 0.9 in patients with modified CHA_2_DS_2_-VASc score of 0, 1, 2, 3, 4, 5, 6, 7, and 8 was 0%, 0.9%, 1.3%, 5.9%, 14.2%, 18.4%, 20.8%, 46.2%, and 50.0%, respectively (p < 0.001). There was no patient with modified CHA_2_DS_2_-VASc score of 9 in our study. Figure 1B shows the percentage of ABI < 0.9 in patients with modified CHA_2_DS_2_-VASc score <2 and ≥2. The percentage of ABI < 0.9 in patients with modified CHA_2_DS_2_-VASc score of <2 and ≥2 was 0.7% and 7.7%, respectively (p < 0.001)

## Discussion

In this study, we evaluated the association of ABI < 0.9 with modified CHA_2_DS_2_-VASc score. We found that advanced age, lower eGFR, and modified CHA_2_DS_2_-VASc score was independently associated with ABI < 0.9. In addition, the percentage of ABI < 0.9 in patients with modified CHA_2_DS_2_-VASc score of 0, 1, and <2 were 0%, 0.9%, and 0.7%, respectively (All < 1%).

CHA_2_DS_2_-VASc score is a validated clinical prediction tool, which outperforms the CHADS_2_ score in predicting stroke and systemic embolism of AF patients^22^. Several recent studies extended the usage of CHA_2_DS_2_-VASc score to non-AF population^30–32^. Mustafa Cetin *et al*. reported that CHA_2_DS_2_-VASc score was increased in patients with mild and severe CAD, and it was correlated significantly with the number of diseased vessels and Gensini score. Therefore, their findings suggested that CHA_2_DS_2_-VASc score might be useful in prediction of the risk of severe CAD^30^. Modi R *et al*. also found that CHADS_2_ score and CHA_2_DS_2_-VASc score correlated significantly with CAD severity and suggested these scores might play an important role in predicting the severity of CAD^31^. In addition, Hoshino T *et al*. reported that CHADS_2_ score > 2 and CHA_2_DS_2_-VASc score > 4 are associated with 3-month functional outcome of stroke in patients with prior coronary artery disease^32^.

Patients with PAOD have significantly increased cardiovascular morbidity and mortality. The 5-year mortality of patients with asymptomatic and symptomatic PAOD is 19% and 24%^8^. However, in patients with critical limb ischemia, mortality rates could be as high as 20% within 6 months from diagnosis and exceeding 50% at 5 years^9^. Therefore, how to identify high risk and low risk patients become extremely important during initial diagnosis. Risk factors of PAOD include advanced age, diabetes, hypertension, stroke, heart failure, and so on^6,10–14^. Advanced age, diabetes, and hypertension were considered as traditional risk factors of PAOD. Because PAOD was a systemic atherosclerotic process and shares similar risk factors to atherosclerosis in the coronary and carotid arteries, there was also a strong association of PAOD with stroke and CAD^11–13,16,17^. Coexistent CAD and stroke were highly prevalent in patients with PAOD particularly in the elderly population^7,17^. Adesunloye BA *et al*. also showed patients with heart failure were over 3 times more likely to have PAOD compared to the general population^14^.

PAOD was more prevalent in women than generally appreciated. The estimates of PAOD varied greatly according to the different diagnostic criteria. PAOD was also be affected by different race^15^. According to a systemic review of global estimates of prevalence and risk factors for PAOD in 2000 and 2010, the association of sex with PAOD had an inconsistent result in the two setting. For all countries, female overall had a significantly higher risk than male, but in high income countries, male had an increased risk of PAOD than females^18^. In our previous nationwide cohort study of PAOD in Taiwan, female gender was found to be a significant predictor of new-onset POAD after multivariate analysis^20^. Because the components of modified CHA_2_DS_2_-VASc score were significantly correlated with PAOD, modified CHA_2_DS_2_-VASc score itself should have a strong correlation with PAOD. In the present study, we consistently demonstrated that modified CHA_2_DS_2_-VASc score was significantly associated with ABI < 0.9 not only in the univariate analysis but also in the multivariate analysis. In addition, although CHADS_2_ score was significantly associated with ABI < 0.9 in the univariate analysis, the association disappeared in the multivariate analysis. Hence, modified CHA_2_DS_2_-VASc score might be more useful in identifying patients with PAOD than CHADS_2_ score.

Although CHADS_2_ score was a useful and simple tool to estimate the risk of PAOD in our previous study^19,20^, it still had the limitation to identify low-risk patients which is similar to its usage in AF patients for stroke and systemic embolism prediction. In our study, the percentage of ABI < 0.9 in patients with CHADS_2_ score of 0 and 1 were 0.7% and 1.7%; however; the percentage of ABI < 0.9 in patients with CHA_2_DS_2_-VASc score of 0 and 1 were 0% and 0.9% which were both <1%. These results suggest that CHA_2_DS_2_-VASc score might be a more useful clinical tool for new-onset PAOD prediction and help physicians to further stratify the risk of PAOD than CHADS_2_ score in non-AF patients.

The prevalence of ABI < 0.9 was 5.6% in our study. According to the previous literature, the prevalence of PAOD in Asia was less than 5% in the general population^33^. In addition, Chen *et al*. also reported that overall prevalence of ABI < 0.9 in 1915 Taiwanese patients was 5.4%, which was also similar to our study^34^.

Although there are several similar risk factors such as age, hypertension, diabetes, and heart failure between CHA_2_DS_2_-VASc score and stroke, CAD, and even PAOD, there still exists many dissimilarities between them. For example, when the predicted outcome is stroke, the definition of vascular disease of CHA_2_DS_2_-VASc score is prior myocardial infarction, PAOD, or aortic plaque. However, when the predicted outcomes is CAD, some studies define the vascular disease as the one used in stroke prediction^35^, but some studies define the vascular disease as PAOD only^30,31^. In the present study, we evaluated the association between PAOD and modified CHA_2_DS_2_-VASc score, so we defined the vascular disease as CAD only.

There were several limitations to our study. First, because our study was a cross-sectional one, we could only confirm the significant association of modified CHA_2_DS_2_-VASc score with ABI < 0.9. We could not elucidate the true cause-effect relationship between them. Second, since the subjects of this study were already being evaluated for heart disease, it was susceptible to selection bias and making findings potentially less generalized. Third, the majority of our patients were treated chronically with antihypertensive medications. For ethical reasons, we did not withdraw these medications and could not exclude the influence of antihypertensive agents on our findings. However, we had adjusted these parameters during multivariate analysis in our study.

In conclusion, our study demonstrated modified CHA_2_DS_2_-VASc score was significantly associated with ABI < 0.9. Calculation of modified CHA_2_DS_2_-VASc score might be useful in identifying patients with PAOD and in stratifying the risk of PAOD in non-AF patients. Future prospective study is needed to examine the ability of modified CHA_2_DS_2_-VASc score in prediction of newly-onset of PAOD.

## Methods

### Study subjects

Our study was a cross-sectional study. Study subjects were consecutively included from a group of patients who were arranged for echocardiographic examinations at Kaohsiung Municipal Hsiao-Kang Hospital. We excluded patients with significant aortic or mitral valve diseases, AF, and inadequate image visualization. Finally, a total of 1482 patients were included.

### Ethics statement

The study methods were carried out in accordance with the approved guidelines. The study protocol was approved by the institutional review board committee of the Kaohsiung Medical University Hospital (KMUH-IRB-20130093). Informed consents have been obtained in written form from patients. All clinical investigation was conducted according to the principles expressed in the Declaration of Helsinki. The patients gave consent for the publication of the clinical details.

### ABI assessment

The values of ABI were measured by an ABI-form device (VP1000; Colin Co. Ltd., Komaki, Japan), which simultaneously and automatically measured blood pressures in both arms and ankles using an oscillometric method^36,37^. The ABI was calculated by the ratio of the ankle systolic blood pressure divided by the higher systolic blood pressure of the arms. After obtaining bilateral ABI values, the lower one was used for later analysis. The ABI measurement was done once in each patient.

### Collection of demographic, medical, and laboratory data

Demographic and medical data including age, gender, smoking history, and comorbid conditions were obtained from interviews or medical records of patients. The body mass index (BMI) was calculated as the ratio of weight in kilograms divided by square of height in meters. Laboratory data such as triglyceride, total cholesterol, and uric acid were measured from fasting blood samples. The value of estimated glomerular filtration rate (eGFR) was calculated using the equation in the Modification of Diet in Renal Disease (MDRD) study^38^. In addition, medications of patients including aspirin, β-blockers, calcium channel blockers, angiotensin converting enzyme inhibitors (ACEIs), angiotensin II receptor blockers (ARBs), diuretics, and statins during the study period were obtained from medical records.

### Assessment of CHADS_2_ score and modified CHA_2_DS_2_-VASc score

The CHADS_2_ score is derived from the sum of point values of individual risk factors: congestive heart failure, hypertension, age ≥ 75 years, diabetes (1 point each), and prior stroke (2 points). The modified CHA_2_DS_2_-VASc score is derived from the sum of point values of individual risk factors: congestive heart failure, hypertension, age between 65 and 74 years, diabetes, female sex, vascular disease except PAOD (1 point each), age ≥ 75 years (2 points), and prior stroke (2 points). Because the aim of current study was to evaluate the risk of PAOD, vascular disease in this study was modified as vascular disease except PAOD. Congestive heart failure was defined as left ventricular systolic dysfunction with left ventricular ejection fraction ≦40% or having a known history of congestive heart failure. Hypertension was defined as systolic blood pressure ≧140 mmHg or diastolic blood pressure ≧90 mmHg or anti-hypertensive drugs were prescribed. Diabetes was defined as fasting blood glucose level greater than 126 mg/dL or hypoglycemic agents were used to control blood glucose levels. Prior stroke was defined as history of cerebrovascular disease including cerebral bleeding and infarction.

### Statistical analysis

The SPSS 18.0 (SPSS, Inc., Chicago, IL, USA) was used for statistical analysis. Data are expressed as percentages or mean ± standard deviation. Categorical and continuous variables between groups were compared by independent Chi-square test and samples t-test, respectively. The relationship between variables and ABI < 0.9 was assessed by univariate regression analysis. Subsequently, significant variables in the univariate analysis were further analyzed by forward multiple logistic regression analysis to identify the parameters associated with ABI < 0.9. All tests were 2-sided, and the level of significance was established as p < 0.05.

## References

[1] Fowkes FG (1991). Edinburgh Artery Study: prevalence of asymptomatic and symptomatic peripheral arterial disease in the general population. Int J Epidemiol.

[2] Hasimu B (2006). Ankle brachial index as a marker of atherosclerosis in Chinese patients with high cardiovascular risk. Hypertens Res.

[3] Heald CL, Fowkes FG, Murray GD, Price JF (2006). & Ankle Brachial Index, C. Risk of mortality and cardiovascular disease associated with the ankle-brachial index: Systematic review. Atherosclerosis.

[4] Hanssen NM (2012). Associations between the ankle-brachial index and cardiovascular and all-cause mortality are similar in individuals without and with type 2 diabetes: nineteen-year follow-up of a population-based cohort study. Diabetes Care.

[5] Chen SC (2010). Ankle brachial index as a predictor for mortality in patients with chronic kidney disease and undergoing haemodialysis. Nephrology (Carlton).

[6] Bartholomew JR, Olin JW (2006). Pathophysiology of peripheral arterial disease and risk factors for its development. Cleve Clin J Med.

[7] Lee WH (2014). Cardiovascular events in patients with atherothrombotic disease: a population-based longitudinal study in Taiwan. PLoS One.

[8] Diehm C (2009). Mortality and vascular morbidity in older adults with asymptomatic versus symptomatic peripheral artery disease. Circulation.

[9] Abu Dabrh, A. M. et al. The natural history of untreated severe or critical limb ischemia. *J Vasc Sur*g **62**, 1642–1651 e1643, 10.1016/j.jvs.2015.07.065 (2015). 10.1016/j.jvs.2015.07.065 26391460

[10] Selvin E, Erlinger TP (2004). Prevalence of and risk factors for peripheral arterial disease in the United States: results from the National Health and Nutrition Examination Survey, 1999–2000. Circulation.

[11] Banerjee A, Fowkes FG, Rothwell PM (2010). Associations between peripheral artery disease and ischemic stroke: implications for primary and secondary prevention. Stroke.

[12] Meves SH (2010). Peripheral arterial disease as an independent predictor for excess stroke morbidity and mortality in primary-care patients: 5-year results of the getABI study. Cerebrovasc Dis.

[13] Topakian R (2010). High prevalence of peripheral arterial disease in patients with acute ischaemic stroke. Cerebrovasc Dis.

[14] Adesunloye BA, Valadri R, Mbaezue NM, Onwuanyi AE (2012). Impact of peripheral arterial disease on functional limitation in congestive heart failure: results from the national health and nutrition examination survey (1999-2004). Cardiol Res Pract.

[15] Mosca L (1997). Cardiovascular disease in women: a statement for healthcare professionals from the American Heart Association. Writing Group. Circulation.

[16] Hirsch AT (2001). Peripheral arterial disease detection, awareness, and treatment in primary care. JAMA.

[17] Shammas NW (2007). Epidemiology, classification, and modifiable risk factors of peripheral arterial disease. Vasc Health Risk Manag.

[18] Fowkes FG (2013). Comparison of global estimates of prevalence and risk factors for peripheral artery disease in 2000 and 2010: a systematic review and analysi*s*. Lancet.

[19] Hsu PC (2014). Association between the CHADS2 score and an ankle-brachial index of 0.9 in patients without atrial fibrillation. J Atheroscler Thromb.

[20] Hsu PC (2015). CHADS2 Score and Risk of New-onset Peripheral Arterial Occlusive Disease in Patients without Atrial Fibrillation: A Nationwide Cohort Study in Taiwan. J Atheroscler Thromb.

[21] Gage BF (2001). Validation of clinical classification schemes for predicting stroke: results from the National Registry of Atrial Fibrillation. JAMA.

[22] Lip GY, Nieuwlaat R, Pisters R, Lane DA, Crijns HJ (2010). Refining clinical risk stratification for predicting stroke and thromboembolism in atrial fibrillation using a novel risk factor-based approach: the euro heart survey on atrial fibrillation. Chest.

[23] Camm AJ (2012). focused update of the ESC Guidelines for the management of atrial fibrillation: an update of the 2010 ESC Guidelines for the management of atrial fibrillation. Developed with the special contribution of the European Heart Rhythm Association. Eur Heart J.

[24] January, C. T. *et al*. AHA/ACC/HRS guideline for the management of patients with atrial fibrillation: executive summary: a report of the American College of Cardiology/American Heart Association Task Force on practice guidelines and the Heart Rhythm Society. Circulation 130, 2071–2104, 10.1161/CIR.0000000000000040 (2014). 10.1161/CIR.0000000000000040 24682348

[25] Kirchhof P (2016). ESC Guidelines for the management of atrial fibrillation developed in collaboration with EACTS. Eur Heart J.

[26] Chiang CE (2016). Guidelines of the Taiwan Heart Rhythm Society and the Taiwan Society of Cardiology for the management of atrial fibrillation. J Formos Med Assoc.

[27] Guo Y (2013). Validation of contemporary stroke and bleeding risk stratification scores in non-anticoagulated Chinese patients with atrial fibrillation. Int J Cardiol.

[28] Chao TF (2016). Comparisons of CHADS2 and CHA2DS2-VASc scores for stroke risk stratification in atrial fibrillation: Which scoring system should be used for Asians?. Heart Rhythm.

[29] Siu CW, Lip GY, Lam KF, Tse HF (2014). Risk of stroke and intracranial hemorrhage in 9727 Chinese with atrial fibrillation in Hong Kong. Heart Rhythm.

[30] Cetin M (2014). Prediction of coronary artery disease severity using CHADS2 and CHA2DS2-VASc scores and a newly defined CHA2DS2-VASc-HS score. Am J Cardiol.

[31] Modi R (2017). CHA2DS2-VASc-HSF score - New predictor of severity of coronary artery disease in 2976 patients. Int J Cardiol.

[32] Hoshino T, Ishizuka K, Shimizu S, Uchiyama S (2014). CHADS2, CHA2DS2-VASc, and R2CHADS2 scores are associated with 3-month functional outcome of stroke in patients with prior coronary artery disease. Circ J.

[33] Liu JH (2010). Peripheral arterial disease and clinical risks in Taiwanese hemodialysis patients. Angiology.

[34] Chen YJ (2014). Prevalence of asymptomatic peripheral arterial disease and related risk factors in younger and elderly patients in Taiwan. Angiology.

[35] Huang FY (2017). CHADS2, CHA2DS2-VASc and R2CHADS2 scores predict mortality in patients with coronary artery disease. Intern Emerg Med.

[36] Yamashina A (2002). Validity, reproducibility, and clinical significance of noninvasive brachial-ankle pulse wave velocity measurement. Hypertens Res.

[37] Tomiyama H (2003). Influences of age and gender on results of noninvasive brachial-ankle pulse wave velocity measurement–a survey of 12517 subjects. Atherosclerosis.

[38] Levey AS (1999). A more accurate method to estimate glomerular filtration rate from serum creatinine: a new prediction equation. Modification of Diet in Renal Disease Study Group. Ann Intern Med.

